# The expression of PD-L1 on tumor-derived exosomes enhances infiltration and anti-tumor activity of αCD3 × αPD-L1 bispecific antibody-armed T cells

**DOI:** 10.1007/s00262-024-03785-4

**Published:** 2024-08-06

**Authors:** Jaewon Cho, Nara Tae, Yujeong Song, Chae-Won Kim, Seung-Joo Lee, Jae-Hee Ahn, Kwang-Ho Lee, Byung-Hyun Lee, Byung Soo Kim, Sun-Young Chang, Dae Hee Kim, Hyun-Jeong Ko

**Affiliations:** 1 Department of Pharmacy, Kangwon National University, Chuncheon, 24341 Republic of Korea; 2 Kangwon Institute of Inclusive Technology, Kangwon National University, Chuncheon, 24341 Republic of Korea; 3 Department of Advanced Material Science and Engineering, College of Engineering, Kangwon National University, Chuncheon, 25561 Korea; 4 Department of Internal Medicine, Korea University College of Medicine, Seoul, 02841 Republic of Korea; 5 Laboratory of Microbiology, College of Pharmacy, and Research Institute of Pharmaceutical Science and Technology (RIPST), Ajou University, Suwon, 16499 Korea; 6 Innovative Drug Development Research Team for Intractable Diseases (BK21 plus), Kangwon National University, Chuncheon, 24341 Republic of Korea; 7 Global/Gangwon Innovative Biologics-Regional Leading Research Center (GIB-RLRC), Kangwon National University, Chuncheon, 24341 Republic of Korea

**Keywords:** Immunotherapy, Bispecific T-cell engager, Programmed death-ligand 1, Exosomal programmed death-ligand 1

## Abstract

Anti-cluster of differentiation (CD) 3 × α programmed death-ligand 1 (PD-L1) bispecific T-cell engager (BsTE)-bound T-cells (BsTE:T) are a promising new cancer treatment agent. However, the mechanisms of action of bispecific antibody-armed activated T-cells are poorly understood. Therefore, this study aimed to investigate the anti-tumor mechanism and efficacy of BsTE:T. The BsTE:T migration was assessed in vivo and in vitro using syngeneic and xenogeneic tumor models, flow cytometry, immunofluorescence staining, transwell migration assays, microfluidic chips, Exo View R100, western blotting, and clustered regularly interspaced short palindromic repeats (CRISPR)/CRISPR-associated protein 9 technology. In murine B16 melanoma, MC38 colon cancer, and human multiple myeloma cells, BsTE:T exhibited superior tumor elimination relative to that of T-cells or BsTE alone. Moreover, BsTE:T migration into tumors was significantly enhanced owing to the presence of PD-L1 in tumor cells and secretion of PD-L1-containing exosomes. Furthermore, increased infiltration of CD44^high^CD62L^low^ effector memory CD8^+^ T-cells into tumors was closely associated with the anti-tumor effect of BsTE:T. Therefore, BsTE:T is an innovative potential anti-tumor therapy, and exosomal PD-L1 plays a crucial role both in vitro and in vivo in the anti-tumor activity of BsTE:T.

**Graphical abstract:**

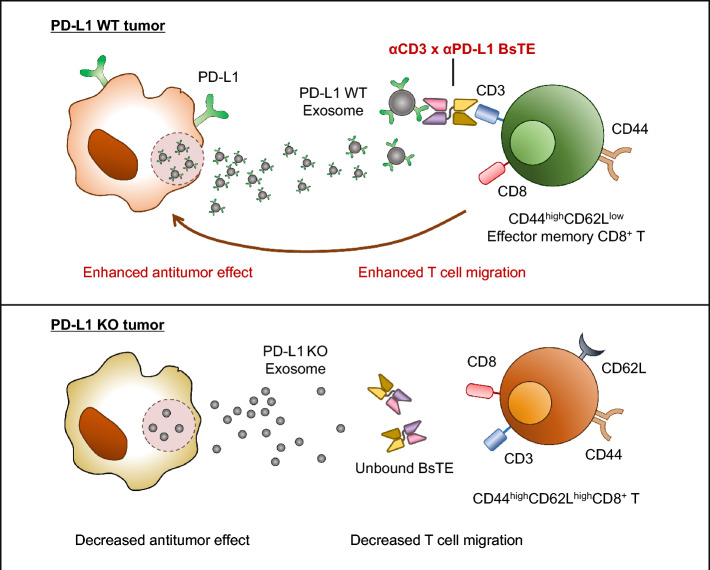

**Supplementary Information:**

The online version contains supplementary material available at 10.1007/s00262-024-03785-4.

## Introduction

Immunotherapeutic agents, such as immune checkpoint inhibitors, and chimeric antigen receptor T (CAR-T) cell therapy, wherein several immune cells exert collaborative anti-cancer activities induced by both modalities, have recently been employed for the treatment of various cancers [1]. However, they also show limited efficacies due to the unfavorable cancer microenvironment [2]. For example, programmed death-ligand 1 (PD-L1) expressed on cancer cells or other cancer stromal cells binds to upregulated PD-1 on activated T-cells, thereby suppressing cancer-killing activity [3]. In the case of CAR-T-cells, virus-transduced antibody-based chimeric receptors are expressed on T-cells and directly exert cancer-killing activities. This may result in an unmanageable activation of several immune cells, leading to side effects such as cytokine release syndrome and neurotoxicity [4]. Bispecific antibody (BsAb), which consists of α cluster of differentiation (CD) 3 and anti-cancer antigen moieties, has been developed as an alternative strategy for CAR T-cell therapy and is currently employed in several clinics [5]. In addition, the strategy of arming activated T-cells (ATC) with targeting αCD3 and tumor-associated antigens (TAA) has been attempted [6]. We previously developed a CD3 and PD-L1-targeting bispecific T-cell engager (αCD3 × αPD-L1 BsTE) and tested its anti-cancer activities in diverse settings using high PD-L1-expressing cells. We found that bispecific T-cell engager (BsTE)-bound activated T-cells (BsTE:T) showed more efficacious anticancer activity than that shown by sole BsTE treatment in syngeneic mouse models [7].

Exosomes are a type of extracellular vesicle generated inside diverse cell types with a diameter of approximately 50–150 nm. They are secreted by cells to deliver intracellular contents to proximal and distal cells [8]. Exosomes contain various biomolecules, such as proteins [8], nucleic acids [9], and metabolites, for cell-to-cell signal transduction [10], with which many cells exchange intracellular information and other types of messages. Cancer cells produce and release exosomes that play important roles in immune regulation within the cancer microenvironment [11]. Moreover, exosomes are associated with organ tropism during cancer cell metastasis, which involves integrin on the surface of cancer cell-derived exosomes [12, 13]. However, only a few studies have investigated the effects of exosomes on immune cell migration [14–16]. Therefore, this study aimed to investigate the anti-tumor mechanism and efficacy of BsTE:T.

## Materials and methods

### Cell lines and culture

B16/F10 melanoma cells expressing ovalbumin (MO5) were obtained from Dr. Kenneth Rock (University of Massachusetts). MC38 colon cancer cells (ATCC, Manassas, VA, purchased in 2006) [17] and Her-2/CT26 colon carcinoma cells expressing human Her-2/*neu* [18] were used for in vitro and in vivo experiments. Both cell lines were maintained in Dulbecco’s modified Eagle medium (DMEM; Invitrogen, Waltham, MA). The mouse myeloma cell line NS-1 was obtained from ATCC and maintained in DMEM. The human multiple myeloma cell line, IM-9, was obtained from the Korean Cell Line Bank (Seoul, Korea). IM-9 cells were maintained in Roswell Park Memorial Institute (RPMI) 1640 medium (Invitrogen). All media were supplemented with 10% fetal bovine serum (Invitrogen) and 1% antibiotic–antimycotic solution (Invitrogen). All cells were maintained in a humidified incubator at 37 °C in a 5% CO_2_ atmosphere. The cell lines were sub-cultured every 2 or 3 days.

### PD-L1-knockout (KO) cell line construction and culture

Using clustered regularly interspaced short palindromic repeats (CRISPR)/CRISPR-associated protein 9, PD-L1 was knocked out in MO5 and MC38 cells to generate PD-L1-KO cell lines by deleting the PD-L1 locus. The *LentiCRISPRv2* vector donated by Feng Zhang (Addgene plasmid #52,961; Addgene, Watertown, MA) was cloned with a guide *PD-L1* (*sgPD-L1*) oligo to delete the *CD274/PD-L1* gene. The cloned *sgPD-L1-LentiCRISPRv2* plasmid was amplified with stbl3™ (Invitrogen) in 50 μg/mL of ampicillin in a Luria–Bertani agar plate. The *sgPD-L1* RNA-containing lentivirus was produced in HEK-293FT-cells (Invitrogen) by co-transfection with 6 μg of *sgPD-L1-LentiCRISPRv2*, 1.5 μg/mL of *pMD2.G,* and 4.5 μg/mL of *psPAX2* using Lipofectamine™ 3000 (Invitrogen) transfection reagent for 3 days. The supernatant containing the lentivirus was collected and concentrated using a Lenti-X™ Concentrator (Takara Bio Inc., Shiga, Japan). The MO5 and MC38 cells were infected with concentrated lentivirus and 6 μg/mL of polybrene (Sigma-Aldrich, St. Louis, MO). Twenty-four hours post-infection, the supernatant was removed, and culture media containing 6 μg/mL of puromycin (Sigma-Aldrich) were added to select cells that were transduced with the lentivirus. The target sequence was amplified using specific primers (Macrogen, Seoul, Korea) and cloned into a T/A cloning vector (Invitrogen) to detect the emergence of the indel sequence. To confirm the indel sequence, the target sequence was amplified using specific primers and cloned into the T/A cloning vector. Plasmids were isolated using the following primer sequences: mCD274_F 5′ ACTAACAGGTGATCCGTTTCCT 3′ and mCD274_R 5′ TCCCAGTACACCACTAACGC 3′.

### Construction of αCD3 × αPD-L1 BsTE

The variable domains of the αPD-L1 antibody were obtained using phage display. The variable domains of mouse αCD3 (clone 145-2C11) and human αCD3 (clone OKT3) antibodies were codon-optimized and synthesized commercially (Macrogen). A (G_4_S_1_)_3_ linker was used to fuse V_L_-V_H_ (αPD-L1) or V_H_-V_L_ (αCD3), and a G4S1 linker was used to fuse the αPD-L1 (V_L_-V_H_) scFv fragment and αCD3 (V_H_-V_L_) scFv fragment via overlap-extension polymerase chain reaction (PCR). Subsequently, PCR fragments were subcloned into the pCEP4 vector (Addgene), allowing for accurate in-frame translation of αCD3 × αPD-L1 BsTE.

For our phage display antibody screening, we used a human naïve antibody library generously provided by LG Life Sciences, a private biotech company in South Korea, through a material transfer agreement. This library was employed to isolate human antibody clones that bind to human PD-L1. The library has a diversity of approximately 1.2 × 10^11^, with each scFv expressed with a c-Myc tag at the C-terminus. The human naïve scFv library was re-amplified to display scFv on phages, and phage pools from each round of panning were produced using ER2738 cells (New England Biolabs). Phage display experiments, including library reamplification and phage panning, were mainly performed following the protocols described in “Phage Display: A Laboratory Manual” [19].

### Animal experiments

Five-week-old female C57BL/6 mice, NOG (NOD.Cg-Prkdc^scid^IL2γg^tm1 Sug^/JicKoat), and BALB/c mice were purchased from KOATECH (Pyeongtaek, Korea). OT-1 mice [C57BL/6-Tg(TcraTcrb)1100Mjb/J] were purchased from The Jackson Laboratory (Bar Harbor, ME) [20]. The mice were maintained in a specific pathogen-free animal facility at Kangwon National University (Chuncheon, Korea). All animal procedures were conducted in accordance with the animal protocol approved by the Institutional Animal Care and Use Committee of Kangwon National University (KW-201007–1 and KW-210826–7). Six-week-old mice were subcutaneously transplanted in the right flank with 2 × 10^6^ MO5, PD-L1-KO MO5, MC38, PD-L1-KO MC38, IM-9, or Her-2/CT26. Tumor volumes were measured using calipers as follows: tumor volume = 1/6π × length (mm) × height (mm) × width (mm). When the tumor volumes reached approximately 100 mm^3^, 2 × 10^5^ CD8^+^ T-cells or BsTE-bound CD8^+^ T-cells were injected into the mice via the tail vein. Human peripheral blood mononuclear cells (PBMCs) were obtained from healthy or patient donors, and their use was approved by the institutional review board of the Kangwon national university medical center (Kangwon, Korea) and the Korea university medical center (Seoul, Korea). The donors provided written informed consent for the use of their tissue samples. All methods were performed in accordance with relevant guidelines and regulations (IRB No. 2018AN0150, IRB No. P01-202108–31-008). Human samples were diluted to a ratio of 1:1 with phosphate-buffered saline (PBS) and layered on Ficoll (Histopaque-1077; Sigma-Aldrich) in a 15-mL conical tube. The samples were centrifuged at 400 × g for 30 min at 37 °C to obtain an opaque interface containing mononuclear cells.

### Cytotoxicity assay

CD8^+^ T-cells were purified from the spleen and lymph nodes of OT-1 mice using a CD8^+^ T-cell isolation kit (MACS; Miltenyi Biotech, Bergisch Gladbach, Germany). Thereafter, the isolated CD8^+^ T-cells were incubated with RPMI 1640 medium supplemented with GlutaMAX™-I (Invitrogen), 10% fetal bovine serum (Gibco, Carlsbad, CA), 10 mM HEPES buffer, 100 μg/mL penicillin/streptomycin, 100 μg/mL gentamycin, 50 μM mercaptoethanol (all from Invitrogen), 5 μg/mL anti-CD3 antibody (clone 145-2C11; BioLegend, San Diego, CA), and 2 μg/mL anti-CD28 antibody (BioLegend). For the cell lysis assay, 1 × 10^4^ target cells (MO5 and PD-L1-KO MO5 cells) were seeded in a 96-well plate and incubated in a humidified incubator at 37 °C in a 5% CO_2_ atmosphere. Subsequently, CD8^+^ T-cells were treated with 0.1 μg/mL BsTE and incubated for 1 h at 37 °C in a 5% CO_2_ atmosphere. After incubation, normal CD8^+^ T-cells and CD8^+^ T-cells treated with BsTE were seeded in a 96-well plate containing the target cells in the same proportions at 37 °C in a 5% CO_2_ atmosphere. After incubation for 6 h, 10 μL of cell-counting kit 8 solution (Cell Counting Kit-8; Dojindo Laboratories, Kumamoto, Japan) was added to each well and incubated for an additional 2 h. The absorbance was measured at 450 nm using a SpectraMax i3 microplate reader (Molecular Devices, San Jose, CA).

### Immunohistochemistry

Immunohistochemical analysis was performed on formalin-fixed, paraffin-embedded sections of the tumor tissue. The tissue sections were cut to a thickness of 5 μm and then prepared for immunohistochemical staining and analysis. The slides were baked at 60 °C for 1 h, deparaffinized with xylene, and rehydrated with graded ethanol. Antigen retrieval was performed by incubating the histological sections in 0.01 M citrate buffer (pH 6.0) at 100 °C for 20–30 min. Afterward, the samples were stained with anti-CD8α (D8A8Y) rabbit mAb (Cell signaling technology, Danvers, MA), hematoxylin, and 3, 3'-diaminobenzidine using the VECTASTAIN® Elite® ABC Universal PLUS Kit and peroxidase (Horse Anti-Mouse/Rabbit IgG; Vector Laboratories, Newark, CA). The stained areas were counted to evaluate the number of CD8^+^ T-cells that infiltrated the tumors.

### Immunofluorescence

Formalin-fixed, paraffin-embedded tumor samples were sectioned into 5-μm-thick sections using a microtome (Leica Biosystems, Wetzlar, Germany). The sections were retrieved by steaming in 10 mM citrate buffer before blocking with bovine serum albumin. Subsequently, the samples were incubated with primary antibodies overnight at 4 °C. To analyze BsTE:T, the following fluorescence-labeled antibodies were used for staining: anti-CD8α-allophycocyanin (APC) (clone 53–6.7; BD Biosciences, Franklin Lakes, NJ) and anti-c-Myc-DyLight 488 (clone 9E10; Novus Biologicals, Centennial, CO). Immunofluorescence (IF) images were obtained using a super-sensitive high-resolution confocal laser scanning microscope (LSM880 with Airyscan, Carl Zeiss, Oberkochen, Germany), at the Central Laboratory of Kangwon National University.

### Migration assay

CD8^+^ T-cell migration was evaluated using a 24-well, 8-μm pore size transwell plate (SPL, Gyeonggi-do, Korea). Briefly, CD8^+^ T-cells or BsTE:T were washed thrice with PBS, resuspended in a T-cell medium at a density of 1 × 10^6^ cells/well, and placed in the apical chamber of the transwell plate. IM-9 or MO5 cells were seeded into the basolateral chamber of the transwell plate as the target cells with the same number of effector cells. PD-L1^+^ or PD-L1^−^ exosomes were added as indicated, and the transwell chambers were incubated for 24 h at 37  °C in a 5% CO_2_ atmosphere. After incubation, the apical chamber was removed, and the number of T-cells that migrated to the bottom well was analyzed using fluorescence-activated cell sorting (FACS). FACS data were obtained at the Core-Facility for Innovative Cancer Drug Discovery (CFICDD) at Kangwon national university.

### Microfluidic chip analysis

The microfluidic chip system was constructed from polydimethylsiloxane (PDMS; Dow Chemical, Midland, MI) using soft lithography with SU-8 (MicroChem, MA). The sterilized PDMS was punched to form two islets, and aisles were carved to connect both islets. The produced PDMS device and cover glass were bonded to form a closed microfluidic channel. Microfluidic chips were autoclaved and dried at 60 °C overnight. Thereafter, a collagen coating solution (50 μg/mL, type I collagen; BD Biosciences) was loaded into the channels to attach the cells, after which the device was washed with sterile water and dried at 60 °C for 24 h. Before using the device for the experiment, type I collagen (BD Biosciences) was used as a scaffold material and gelled between the channels in an incubator at 37 °C for 1 h. The collagen solution was adjusted to pH 7.

MO5 cells (target cells) and CD8^+^ T-cells or BsTE:T (effector cells) were seeded in each channel of the microfluidic chip. Both cell lines were seeded at a density of 5 × 10^4^ cells/well and incubated for 48 h at 37 °C in a 5% CO_2_ incubator. After incubation, both channels were washed with PBS and stained with anti-CD8α-APC (clone 53–6.7; BD Biosciences) and anti-CD3-fluorescein isothiocyanate (FITC) (clone 145-2c11; BD Biosciences).

#### Exosome analysis

To obtain exosomes, 5 × 10^5^/mL of MO5 or PD-L1-KO MO5 cells were seeded and incubated for 24 h. After incubation, or when the cells covered the plate at a rate higher than 70%, supernatant samples were replaced with precondition media containing 10 μg/mL of penicillin/streptomycin in DMEM (Invitrogen) and incubated for 24 h. Thereafter, the supernatant was harvested and centrifuged at 10,000 × *g* for 30 min to obtain a supernatant. To concentrate the exosomes, the supernatant was centrifuged at 42,000 rpm for 70 min, after which the pellet was resuspended in PBS or cell culture medium. The exosomes were isolated using a size-exclusion chromatography kit (EXoPERT Inc., Seoul, Republic of Korea) and stained with the following antibodies: anti-human CD274-APC (clone MIH3; BioLegend), anti-human CD9-FITC (clone HI9a; BioLegend), and anti-human CD81-phycoerythrin (clone 5A6; BioLegend).

#### Transmission *electron* microscopy

Concentrated exosome preparation (5 μL) samples were fixed with an equal volume of 4% paraformaldehyde and 0.1% glutaraldehyde at 4 °C for 1 h and then placed on a carbon-coated 300-mesh copper grid. The exosome morphology was visualized using a Tecnai T10 low-voltage bio-transmission electron microscope (FEI Company, Hillsboro, OR) operated at 100 kV at the Chuncheon Center of Korea Basic Science Institute.

#### Statistical analysis

Statistical analyses were performed using GraphPad Prism, version 9 (GraphPad Software LLC, San Diego, CA). An unpaired two-tailed Student's *t*-test was used to compare differences between two groups when the variables had a Gaussian distribution with similar variances. One-way analysis of variance was used to compare more than two groups, followed by a post-hoc Bonferroni’s test. The threshold for statistical significance was set at *p*-value < 0.05, with 95% confidence intervals estimated for all analyses.

## Results

### Binding of BsTE on CD8 T-cells increased T-cell tumor infiltration

**Fig. 1:**
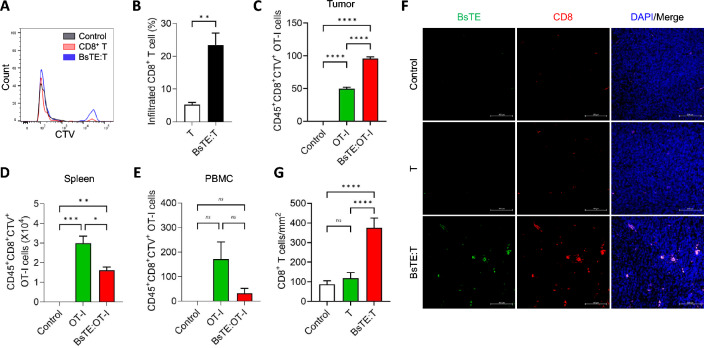
Infiltration of bispecific T-cell engager (BsTE)-bound cluster of differentiation (CD) 8 T-cells into tumors increases at a higher rate than that when using CD8 T-cells alone. (A, B) NS-1 cells (2 × 10^6^ cells/mouse) were transplanted to the left flank of C57BL/6 mice, and T-cells or BsTE:T (2 × 10^5^ cells/mouse) stained with cell trace violet (CTV) were intravenously injected when the tumor size reached 100 mm^3^. Twenty-four hours after the injection of T-cells or BsTE:T, the mice were euthanized, and tumors were isolated and analyzed using CTV^+^CD8^+^ T-cells through flow cytometry. **p < 0.01 (Student’s *t*-test; *n* = 3/group). (C–E) Groups of mice were subcutaneously injected with MO5 cells (2 × 10^6^ cells/mouse) in their left flank, and when the tumor grew sufficiently, OT-1 cells or BsTE:OT-1 cells (2 × 10^5^ cells/mouse) were injected into the mice via the tail vein. Twenty-four hours after the injection of OT-1 or BsTE:OT-1 cells, mice were euthanized, and the spleen, peripheral blood mononuclear cells, and tumor samples were prepared for flow cytometry analysis. Ns; not significant, *p < 0.05, **p < 0.01, ***p < 0.001, ****p < 0.0001 (one-way analysis of variance [ANOVA]; *n* = 3/group). (F, G) Representative immunofluorescence (IF) images of MO5 tumors (× 200). T-cells or BsTE:T (2 × 10^5^ cells/mouse) were intravenously injected, and after 24 h, the mice were euthanized. Tumor samples were prepared for IF, and BsTE (anti-c-Myc; DyLight 488), CD8 (anti-CD8α; allophycocyanin [APC]), and 4′,6-diamidino-2-phenylindole (DAPI) were stained to evaluate T-cell infiltration. Ns; not significant, ****p < 0.0001 (one-way ANOVA; *n* = 32/group) Scale bar: 100 µm

In a previous study, we developed a strategy for enhancing anti-tumor effects that involved combining mouse αCD3 × αPD-L1 BsTE:T [7]. Our investigation using cell trace violet (CTV) vital dye-labeled CD8^+^ T-cells revealed that BsTE:T exhibited higher infiltration into tumors compared with that of T-cells alone, as indicated by the presence of CTV-positive cells in the tumors 24 h post-injection (Fig. 1A, B). Flow cytometry analysis of CD45^+^CD8^+^CTV^+^OT-1 cells in MO5-bearing mice at the PBMC, spleen, and tumor sites 24 h after the injection of OT-1 and BsTE-bound OT-1 cells showed similar results. The infiltration of OT-1 cells pre-bound with BsTE was significantly greater in the tumor compared with that in the spleen and PBMCs, whereas administering OT-1 cells alone did not result in enhanced infiltration in the mouse tumor (Fig. 1C–E).

Moreover, the infiltration of BsTE:T by directly detecting BsTE and T-cells was assessed. After validating the cMyc-tag on the αCD3 × αPD-L1 BsTE construct (Supplementary Fig. 1). IF staining was conducted to confirm BsTE:T infiltration. Furthermore, the engagement of BsTE on T-cells alone did not activate T-cells and anti-CD3/CD28 antibody treatment for T-cell activation did not downregulate CD3 expression on activated T-cells (Supplementary Fig. 2A–C), suggesting that BsTE can bind to the surface of activated T-cells. IF staining of tumor tissues from mice injected with T-cells alone or with BsTE:T was conducted to directly detect BsTE:T and BsTE molecules in the tumors. CD8^+^ T-cells pre-treated with BsTE showed increased infiltration, and the BsTE molecules colocalized well with CD8^+^ T-cells in the BsTE:T-treated mice (Fig. 1F). Meanwhile, the control mice had fewer CD8^+^ T-cell infiltrations in the tumors than those in the BsTE:T-treated mice (Fig. 1F, G). Collectively, we confirmed that BsTE binding enhances the infiltration of T-cells into the tumor, and the infiltrated T-cells can be found in a BsTE-bound form in the tumor tissue.

### Enhanced T-cell migration of BsTE:T was dependent on tumor PD-L1 expression

**Fig. 2:**
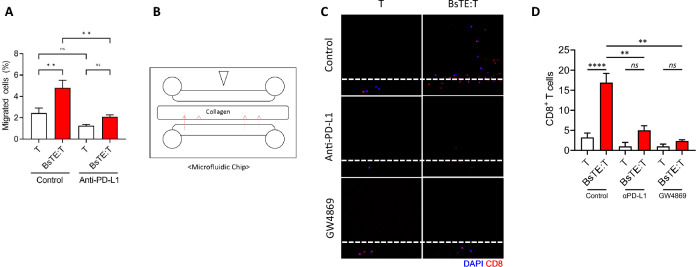
Migration of bispecific T-cell engager-bound T-cells (BsTE:T) is inhibited by Αpd-L1 or GW4869 treatment. (A) T-cells or BsTE:T were activated with anti-cluster of differentiation (CD)3/CD28 antibodies for 48 h, and bispecific T-cell engager (BsTE; 0.1 μg/mL) was incubated for 1 h. T-cells or BsTE:T were seeded in transwell plates containing pores to allow cell migration; MO5 cells were seeded at the bottom of the transwell. After 24 h, all cells in the basolateral transwell were extracted for analysis by flow cytometry. Ns; not significant, **p < 0.01 (one-way analysis of variance [ANOVA]; *n* = 5/group). **B** Diagram of the microfluidic chip. The microfluidic chip contains three semipermeable layers. The middle layer was filled with collagen, and different cells were seeded within the other two layers. **C**, **D** The microfluidic chips were designed with one collagen layer, which surrounded two semipermeable layers, and effector cells were seeded in one layer, whereas target cells were seeded in the other layer. Every T-cell that infiltrated the collagen layer was counted. CD8 (anti-CD8α; allophycocyanin [APC]) and 4′,6-diamidino-2-phenylindole (DAPI) were stained. Ns; not significant, **p < 0.01 and ****p < 0.0001 (Student’s *t*-test; *n* = 9/group)

To investigate the mechanism underlying enhanced BsTE:T migration into tumors, we conducted an in vitro transwell migration assay. The enhanced migration of BsTE:T toward the MO5 cell-containing bottom well was observed in contrast to sole T-cell seeding, and PD-L1 antibody treatment in the bottom well abrogated the migration of BsTE:T to MO5 cells (Fig. 2A), demonstrating the potential role of PD-L1 in T-cell migration to tumor cells. Similarly, the association of BsTE:T migration with PD-L1 expression was confirmed using a microfluidic chip filled with collagen between two semipermeable layers (Fig. 2B). MO5 cells were seeded on one side and T-cells or BsTE:T were seeded on the other side of the semipermeable layer. After 24 h of incubation, fluorescence staining with 4′,6-diamidino-2-phenylindole and anti-CD8 antibodies in the collagen layer revealed a significant number of BsTE:T infiltrating the collagen layer, whereas most T-cells remained non-infiltrated in the semipermeable layer (Fig. 2C). The number of CD8^+^ T-cells infiltrating the collagen layer was counted using ZEN software (Carl Zeiss) (Fig. 2D). Considering that the addition of αPD-L1 Ab inhibited the infiltration of BsTE:T into the collagen layer (Fig. 2C), BsTE-mediated T-cell migration seemed to be dependent on PD-L1 function. The collagen layer effectively separated MO5 cells from T-cells or BsTE:T in our microfluidic chip, indicating that a direct interaction between these two cell layers was unlikely. Thus, we hypothesized that specific tumor-derived materials, potentially containing PD-L1, such as extracellular vesicular materials originating from tumor cells, may influence the migration of BsTE:T. To confirm whether BsTE-mediated T-cell migration is dependent on exosomes, small extracellular vesicle and exosome secretion by MO5 cells was blocked using GW4869, a neutral sphingomyelinase inhibitor and widely used pharmacological agent for blocking exosome generation [21]. The number of infiltrating BsTE:T was dramatically reduced after GW4869 treatment (Fig. 2C, D). These results suggest that the increased infiltration of BsTE-conjugated T-cells depends on the presence of tumor-derived exosomes that may contain PD-L1.

### PD-L1-containing exosomes enhanced BsTE:T migration to tumor tissues

**Fig. 3:**
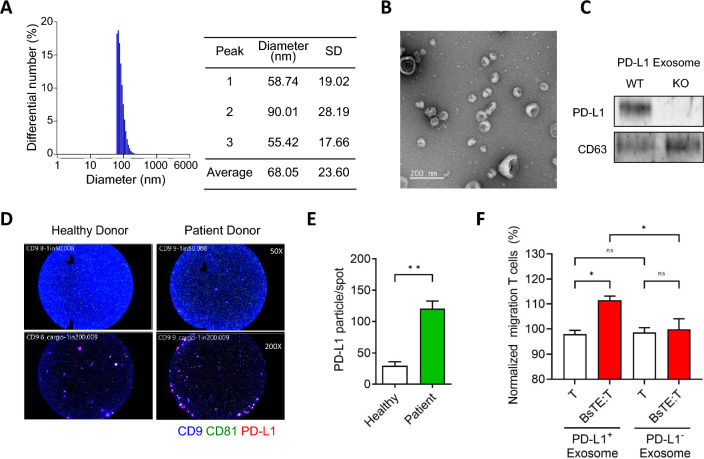
Programmed death-ligand 1 (PD-L1) present in exosomes helps bispecific T-cell engager-bound T-cells (BsTE:T) migrate into tumor tissue. (A) Dynamic light scattering and laser Doppler methods (ELS-Z1000; Otsuka Electronics, Tokyo, Japan) were used to measure the diameter of exosomes that were obtained from MO5 cells. **B** Isolated exosome images were obtained using transmission electron microscopy, allowing measurement of exosome diameter. Scale bar: 200 nm. **C** Isolated exosomes from wild type (WT) and PD-L1-knockout (KO) B16 cell culture supernatant were detected by the αPD-L1 antibody through western blotting. Exosome existence was confirmed by cluster of differentiation (CD)63. **D**, **E** ExoView R100 was used to determine the exosomes stained with CD9 (anti-CD9; pacific blue), CD81 (anti-CD81; fluorescein isothiocyanate [FITC]), and CD274 (anti-human CD274 or B7-H1, PD-L1; allophycocyanin [APC]). Ns; not significant, and **p < 0.01 (Student’s *t*-test; *n* = 3/group). **F** T-cells were obtained from the spleen or mesenteric lymph node of C57BL/6 mice and activated with anti-CD3/CD28 antibodies for 48 h, and BsTE:T were incubated with BsTE for 1 h. After the preparation of T-cells or BsTE:T, both cells were seeded in transwell plates, and PD-L1-KO MO5 cells were seeded in the basolateral transwell. PD-L1^+^ or PD-L1^−^ exosomes were treated with PD-L1-KO MO5 cells. After transwell incubation for 24 h, cells were extracted from the basolateral transwell and analyzed using fluorescence-activated cell sorting (FACS). Ns; not significant and *p < 0.05 (one-way analysis of variance [ANOVA]; *n* = 3/group). SD, standard deviation

After confirming the involvement of exosomes and PD-L1 molecules from cancer cells in the migration of BsTE:T to cancer cells, we sought to confirm whether exosomes possess PD-L1 molecules. Exosomes from the culture supernatants of MO5 cells were harvested and characterized in terms of size, morphology, and PD-L1 incorporation. The diameters of exosomes from MO5 cells measured by dynamic light scattering and laser Doppler methods were 68.05 nm (standard deviation, 23.60 nm), on average, consistent with previously reported estimates (approximate range of 30 to 160 nm) (Fig. 3A). Exosome morphology was analyzed using transmission electron microscopy, which confirmed that they were small membrane-bound vesicles (Fig. 3B). Next, we isolated exosomes from the culture supernatants of wild-type (WT) and PD-L1-KO MO5 cells to investigate PD-L1 presence. PD-L1 was only detected in exosomes isolated from the WT MO5 cell culture supernatant, whereas exosomes isolated from the PD-L1-KO MO5 cancer cell culture supernatant did not show any evidence of PD-L1 (Fig. 3C).

Furthermore, exosomal PD-L1 expression in samples from patients with multiple myeloma was analyzed using ExoView R100 (Accela, San Ramon, CA), an exosome characterization system. The results revealed a higher level of PD-L1 expression in patient samples than that in healthy donors (Fig. 3D), and significant differences in the surface-expressed PD-L1 levels were observed (Fig. 3E). Thus, we found that PD-L1 was expressed on exosomes and that the level of exosomal PD-L1 expression was higher in the plasma of patients with multiple myeloma than that in the plasma of healthy donors.

To investigate the role of PD-L1 molecules in the migration of BsTE:T, PD-L1-expressing (PD-L1^+^) exosomes from MO5 cells and PD-L1-non-expressing (PD-L1^−^) exosomes from PD-L1-KO MO5 cells were prepared, and their effect on BsTE:T migration was evaluated. PD-L1-KO MO5 cells were seeded at the bottom of the transwell system, and PD-L1^+^ or PD-L1^−^ exosomes were added to the bottom, whereas T-cells or BsTE:T were seeded in the upper wells to verify the migration of those cells to the bottom wells. Thus, in the PD-L1^+^ exosome-treated group, BsTE:T migrated at a higher rate through the transwell relative to T-cells, whereas the PD-L1^−^ exosome-treated group showed no differences in migration rates between T-cells and BsTE:T (Fig. 3F). Furthermore, the number of infiltrating BsTE:T in the transwell system was significantly reduced by GW4869 treatment (Supplementary Fig. 3). Collectively, these results suggest that PD-L1^+^ exosomes are key molecules in enhancing the migration of BsTE:T to the tumor.

### BsTE:T anti-tumor activity was highly dependent on in vivo PD-L1 expression

**Fig. 4:**
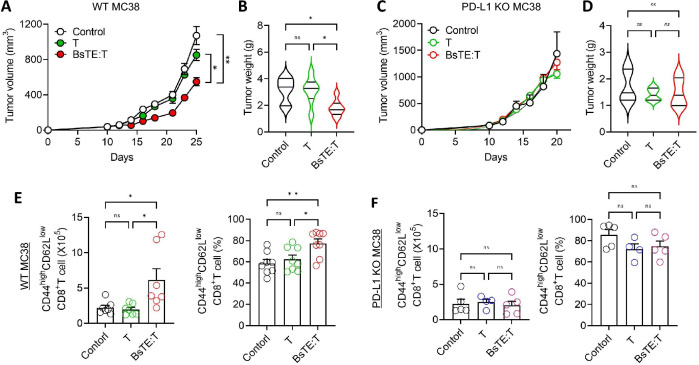
Programmed death-ligand 1 (PD-L1) expression in tumor cells is crucial for the anti-tumor effect of bispecific T-cell engager-bound T-cells (BsTE:T). **A**, **C** MC38 and PD-L1-knockout (KO) MC38 (2 × 10^6^ cells/mouse) were transplanted into the left flank of C57BL/6 mice, and T-cell or BsTE:T (2 × 10^5^ cells/mouse) were intravenously injected when the tumor size reached 100 mm^3^. Tumor size was measured every 2 days and monitored for 20 or 25 days. ns; not significant, *p < 0.05, and **p < 0.01 (one-way analysis of variance [ANOVA]; *n* = 4/MC38, *n* = 5/PD-L1-KO MC38). (B, D) Tumor weight was measured after the mice were euthanized. ns; not significant, and *p < 0.05 (one-way ANOVA; *n* = 7/MC38, *n* = 5/PD-L1-KO MC38). (E, F) The number and population of CD3^+^CD8^+^CD44^high^CD62L.^low^ effector memory T-cells in tumor-infiltrating lymphocytes of every group of mice were analyzed using fluorescence-activated cell sorting (FACS). ns; not significant, *p < 0.05, and **p < 0.01 (one-way ANOVA; *n* = 8/MC38, *n* = 5/PD-L1-KO MC38)

To verify that the enhanced anti-tumor activity of BsTE:T was dependent on PD-L1 expression in tumor cells, PD-L1-KO MC38 cells were prepared, and the anti-tumor efficacy of BsTE:T in vivo was investigated in comparison with that of WT MC38 cells. WT MC38 and PD-L1-KO MC38 cells were subcutaneously inoculated into the left flank of C57BL/6 mice, and after 10 days, T-cells or BsTE:T were intravenously injected into these mice, and tumor sizes were measured every 2 days until the tumor volume reached 1,000 mm^3^. The results for the mice bearing WT MC38 tumors showed obvious BsTE:T anti-tumor activity (Fig. 4A) and decreased tumor weights (Fig. 4B), as expected. However, the size or weight of tumors among the control, T-cell-only, and BsTE:T-treated mice inoculated with PD-L1-KO MC38 cells did not significantly differ (Fig. 4C, D). Furthermore, as observed in the tumor-infiltrated T-cells of BsTE:OT-1 T-cells (Fig. 1E), the tumor-infiltrating lymphocyte analysis of the WT MC38-bearing mice showed that BsTE:T treatment enhanced the accumulation of CD44^high^ CD62L^low^ effector memory CD8^+^ T-cells in the tumor tissue compared with that of central memory CD8^+^ T-cells (CD44^high^ CD62L^high^), whereas control and T-cell-treated mice did not show any significant increase in the level of CD8^+^ T-cell infiltration (Fig. 4E, Supplementary Fig. 4A). Thus, the infiltration of effector memory CD8^+^ T-cells appears to be strongly correlated with anti-tumor efficacy in vivo (Fig. 4E). For mice inoculated with PD-L1-KO MC38 cells, CD8^+^ T-cell infiltration was relatively less frequent, and no notable differences in the infiltration of CD8^+^ T-cells from control, T-cell-only, and BsTE:T-treated mice were observed (Fig. 4F, Supplementary Fig. 4B).

### BsTE:T treatment induced immune activation both in vivo and in vitro, relying on cellular PD-L1 expression and the presence of PD-L1-containing exosomes

**Fig. 5:**
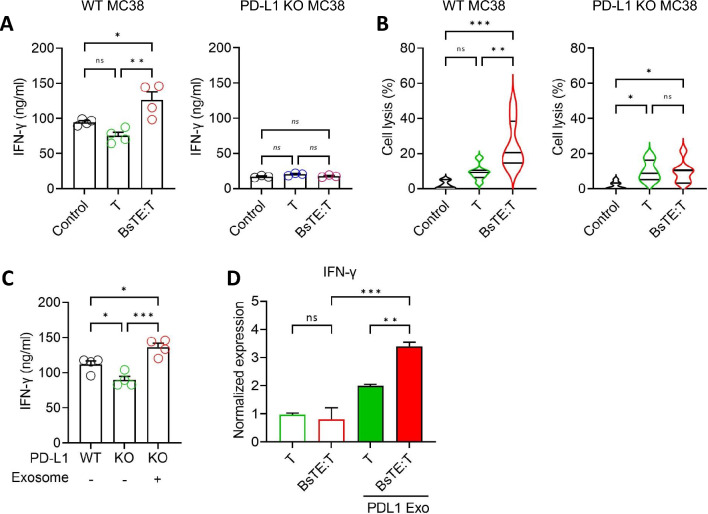
**Antitumor immune responses induced by bispecific T-cell engager-bound T-cells (BsTE:T) mediated by tumor-derived programmed death-ligand 1 (PD-L1)-containing exosomes.** **A** Serum samples from tumor-transplanted mice were obtained from euthanized mice, and interferon (IFN)-γ levels were measured using enzyme linked immunosorbent assay (ELISA). ns; not significant, *p < 0.05, and **p < 0.01 (one-way [ANOVA]; *n* = 4/group). **B** T-cells or BsTE:T (2 × 10^4^ cells/well) were incubated with MC38 or PD-L1-knockout (KO) MC38 cells (2 × 10^4^ cells/well) for 6 h. Cell lysis was measured using a cell counting kit 8 (CCK8) assay. ns; not significant, *p < 0.05, **p < 0.01, and ***p < 0.001 (one-way ANOVA; *n* = 7/group). **C** To measure the secreted IFN-γ from BsTE:T, the supernatant obtained was co-cultured with MO5, PD-L1-KO MO5, or PD-L1^+^ exosome-treated PD-L1-KO MO5 for 24 h. IFN-γ levels were measured using ELISA. ns; not significant, *p < 0.05, and ***p < 0.001 (one-way ANOVA; *n* = 3/group). **D** CD8^+^ T-cells or BsTE:T were co-cultured with PD-L1-KO MO5 or PD-L1^+^ exosome-treated PD-L1-KO MO5 for 24 h. After incubation, CD8.^+^ T-cells or BsTE:T were analyzed using reverse transcription polymerase chain reaction (RT-PCR) to evaluate the level of IFN-γ. ns; not significant, **p < 0.01, and ***p < 0.001 (Student’s t-test; *n* = 3/group)

Immune activation by anti-tumor BsTE:T treatment in WT MC38-transplanted mice was evaluated by measuring serum interferon-gamma (IFN-γ) levels, which were stimulated by BsTE:T (Fig. 5A). No notable immune activation was observed in the mice transplanted with PD-L1-KO MC38 cells (Fig. 5A). PD-L1-dependent tumor-killing activity was also confirmed by in vitro cell lysis assays using WT MC38 or PD-L1-KO MC38 cells, wherein only BsTE:T co-incubated with WT MC38 cells achieved successful cell lysis (Fig. 5B).

The effects of exosomal PD-L1 in the activation of BsTE:T were investigated by measuring IFN-γ levels in the in vitro co-culture of tumor cells with BsTE:T. The co-culture of PD-L1-KO MO5 and BsTE:T induced lower IFN-γ secretion than that of co-culture with WT MO5 and BsTE:T. However, when exosomes isolated from WT MO5 cells (PD-L1^+^) were added to the co-culture of PD-L1-KO MO5 cells, they regenerated BsTE:T activation and produced IFN-γ from T-cells (Fig. 5C). Similarly, when this co-culture of PD-L1-KO MO5 cells was applied with or without BsTE molecules, neither T-cell group was activated enough to produce IFN-γ (Fig. 5D). However, when PD-L1^+^ exosomes were added to the co-cultures, IFN-γ production increased and co-culture with BsTE:T and PD-L1^+^ exosomes showed the highest level of IFN-γ production (Fig. 5D). Collectively, these results suggest that BsTE:T exhibits anti-tumor activity in a PD-L1-dependent manner through enhanced T-cell trafficking into the tumor, which is influenced by PD-L1^+^ exosomes. Specifically, the infiltration of BsTE:T into PD-L1-positive tumors is mediated by tumor-secreted PD-L1^+^ exosomes.

### Combining human BsTE with human PBMCs elicited anti-tumor effects against human multiple myeloma in a cell line-derived xenograft model

**Fig. 6:**
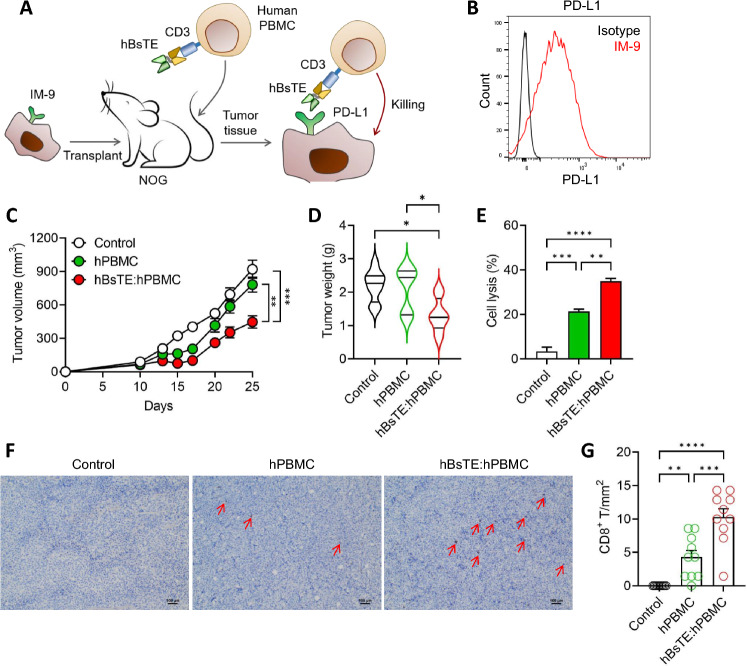
**Enhanced tumor infiltration and anti-tumor efficacy of human bispecific T-cell engager-bound human peripheral blood mononuclear cells (hBsTE:hPBMC) treatment in a human multiple myeloma xenograft model.** **A** Schematic representation of the in vivo experiment using hBsTE:hPBMCs. Groups of mice were transplanted with IM-9 (2 × 10^6^ cells/mouse) into their left flanks and monitored for 25 days. Both human peripheral blood mononuclear cells (hPBMC) and hBsTE:hPBMCs (2 × 10^5^ cells/mouse) were intravenously transferred to tumor-bearing mice 10 days after tumor transplantation. **B** Programmed death-ligand 1 (PD-L1) expression in IM-9 cells. **C** Tumor size was monitored every 2 days. **p < 0.01, ****p < 0.0001 (Student’s *t*-test; *n* = 11/control, *n* = 10/hPBMCs, and *n* = 12/hBsTE:hPBMCs). **D** Tumor weights were measured on day 25 after euthanasia. ns, not significant; *p < 0.05 (one-way analysis of variance [ANOVA]; *n* = 8/group). **E** Human PBMCs were activated using Dynabead human T-activator cluster of differentiation (CD)3/CD28 for 48 h, and the hBsTE (0.1 μg/mL) samples were incubated for 1 h before the cell counting kit 8 (CCK8) assay for cytotoxicity against IM-9 cells. Each cell line was seeded at 1 × 10^4^ cells/well and co-cultured for 2 h. **p < 0.01, ***p < 0.001, and ****p < 0.0001 (one-way ANOVA; *n* = 3/group). **F**, **G** Representative immunohistochemistry images of IM-9 tumors on day 25 (× 100). Arrows indicate infiltrating human CD8^+^ T-cells. CD8^+^ T-cells in each group. **p < 0.01, ***p < 0.001, and ****p < 0.0001 (one-way ANOVA; *n* = 10/group). Scale bar: 100 µm

To expand our research from murine to human cancer models, we developed a human BsTE (hBsTE) capable of engaging human T-cells with PD-L1-positive human cancer cells (Supplementary Fig. 5). NOG mice deficient in T, B, and natural killer cells were transplanted with IM-9 cells (2 × 10^6^ cells/mouse), a human B-lymphoblast-cell line originating from human multiple myeloma (Fig. 6A), which exhibited PD-L1 expression on its surface relative to the isotype antibody (Fig. 6B). Ten days after initial tumor cell transplantation, 2 × 10^5^ hPBMCs or hBsTE-treated hPBMCs (hBsTE:hPBMCs) were intravenously administered into IM-9-bearing mice to evaluate their anti-tumor efficacy. Mice administered hBsTE:hPBMCs displayed a significant decrease in tumor size compared with those in the control and hPBMC-treated groups (Fig. 6C). In addition, given that tumor weight was measured on the day of mouse sacrifice, the hBsTE:hPBMC group showed the least tumor growth compared with the other groups (Fig. 6D). To verify that hBsTE enhanced the cancer-killing activity of hPBMCs, an in vitro killing assay was conducted using IM-9 cells. The hBsTE:hPBMCs showed higher tumor-killing activity against IM-9 cells than hPBMCs alone (Fig. 6E). Furthermore, immunohistochemical analysis showed that hBsTE:hPBMC treatment yielded a higher infiltration of CD8^+^ T-cells into tumor tissues than hPBMC treatment alone (Fig. 6F, G). These results suggest that hBsTE engagement with hPBMCs facilitates the infiltration of human CD8^+^ T-cells into tumor tissues and enhances the killing activity of T-cells.

### Combining PD-L1 BsTE with CAR-T induced effective anti-tumor effects in cancer models

**Fig. 7:**
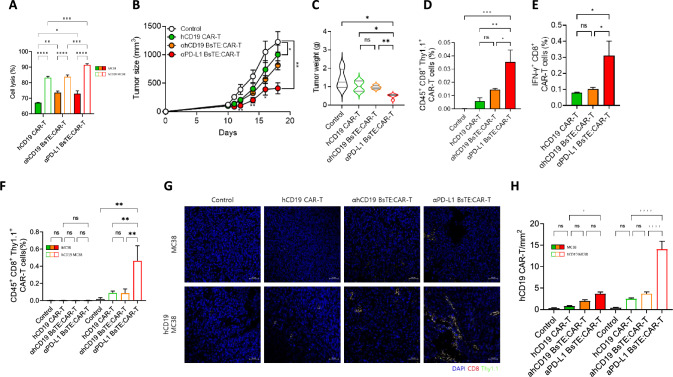
**Enhanced tumor infiltration and anti-tumor efficacy of anti-PD-L1 bispecific T-cell engager-bound chimeric antigen receptor T cell therapy in hCD19 MC38 xenograft models.** **A** Chimeric antigen receptor T cells targeting human CD19 (hCD19 CAR-Ts) were activated with anti-CD3/CD28 antibodies for 48 h, and αhCD19 BsTE or αPD-L1 BsTE (0.1 μg/mL) samples were incubated for 1 h before the cell counting kit 8 (CCK8) assay for cytotoxicity against MC38 and MC38 expressing human CD19. Each cell line was seeded at 1 × 10^4^ cells/well and co-cultured for 2 h. *p < 0.05, **p < 0.01, ***p < 0.001, and ****p < 0.0001 (one-way ANOVA; *n* = 6/group). **B** Groups of mice were transplanted with hCD19 MC38 (2 × 10^6^ cells/mouse) into their left flanks and monitored for 18 days. Groups of hCD19 CAR-T, αhCD19 BsTE:CAR-T, and αPD-L1 BsTE:CAR-T were intravenously transferred effector cells (2 × 105 cells/mouse) to tumor-bearing mice 10 days after tumor transplantation. Tumor size was monitored every 2 days. *p < 0.05 and **p < 0.01 (Student’s *t*-test; *n* = 5/group). **C** Tumor weights were measured on day 18 after euthanasia. ns, not significant; *p < 0.05, and **p < 0.01 (one-way ANOVA; *n* = 5/group). **D** The populations of CD45^+^CD8^+^Thy1.1^+^ CAR-T cells in tumor-infiltrating lymphocytes of every group of mice were analyzed using fluorescence-activated cell sorting (FACS). ns; not significant, *p < 0.05, **p < 0.01, and ***p < 0.001 (one-way ANOVA; *n* = 5/group). **E** The populations of IFN-γ^+^CD8^+^Thy1.1^+^ CAR-T cells in tumor-infiltrating lymphocytes of every group of mice were analyzed using FACS. ns; not significant and *p < 0.05 (one-way ANOVA; n = 5/group). **F** Groups of mice were transplanted with MC38 and hCD19 MC38 (2 × 10^6^ cells/mouse). MC38 were transplanted into their left flank, and hCD19 MC38 were right flank. Groups of hCD19 CAR-T, αhCD19 BsTE:CAR-T, and αPD-L1 BsTE:CAR-T were intravenously transferred to tumor-bearing mice 10 days after tumor transplantation. The populations of CD45^+^CD8^+^Thy1.1^+^ CAR-T cells in tumor-infiltrating lymphocytes of every group of mice were analyzed using FACS. ns, not significant; *p < 0.05, and **p < 0.01 (one-way ANOVA; *n* = 5/group). **G**, **H** Representative immunofluorescence images of MC38 and hCD19 MC38 tumors on day 25 (× 100). Arrows indicate infiltrating DAPI^+^CD8^+^Thy1.1^+^ CAR-T cells. CAR-T cells in each group. ns, not significant; *p < 0.05 and ****p < 0.0001 (one-way ANOVA; n = 50/group). Scale bar: 50 µm

To extend the application of BsTE to the CAR-T cell system, we generated BsTE-bound human CD19 CAR-T (hCD19 CAR-T) cells. Furthermore, to confirm the role of targeting not only PD-L1 BsTE, but also another tumor-associated molecule BsTE, such as αPD-L1 BsTE, we generated αhCD19 BsTE. Therefore, we generated αhCD19 BsTE-bound hCD19 CAR-T (αhCD19 BsTE:CAR-T) and αPD-L1 BsTE-bound hCD19 CAR-T cells (αPD-L1 BsTE:CAR-T). To verify that both αPD-L1 BsTE and αhCD19 BsTE enhanced the cancer-killing activity of hCD19 CAR-T cells, an in vitro killing assay was conducted using both MC38 and MC38 expressing hCD19 (hCD19 MC38). The hCD19 CAR-T showed upregulated killing activity in hCD19 MC38 compared with that in MC38. Based on the cell lysis in hCD19 CAR-T, αhCD19 BsTE:CAR-T showed no significant differences, but αPD-L1 BsTE:CAR-T showed enhanced killing activity against hCD19 MC38 (Fig. 7A). Moreover, C57BL/6 mice were transplanted with hCD19 MC38 (2 × 10^6^ cells/mouse) to expand the investigation of enhancing CAR-T cells. After 10 days from transplanting the tumor cell, 1 × 10^6^ hCD19 CAR-T, αhCD19 BsTE:CAR-T or αPD-L1 BsTE:CAR-T cells were intravenously administered into hCD19 MC38-bearing mice. Mice administered with both hCD19 CAR-T and αhCD19 BsTE:CAR-T showed significant differences in tumor size compared with the control group. Specifically, αPD-L1 BsTE:CAR-T showed significantly decreased tumor size compared with the other groups (Fig. 7B). Given that tumor weight was measured on the day of mouse sacrifice, the αPD-L1 BsTE:CAR-T displayed the least tumor growth compared with the other groups (Fig. 7C). Furthermore, considering that tumor-infiltrated T-cells of BsTE:OT-1 T-cells (Fig. 1E) or hBsTE:hPBMC (Fig. 6F, G) were enhanced because of αPD-L1 BsTE, the tumor-infiltrating hCD19 CAR-T cell analysis of the hCD19 MC38-bearing mice showed that αPD-L1 BsTE:CAR-T (CD45^+^CD8^+^Thy1.1^+^) cell treatment enhanced the accumulation of hCD19 CAR-T cells in the tumor tissue when compared with the control group and hCD19 CAR-T and αhCD19 BsTE:CAR-T cell treatment (Fig. 7D). Additionally, immune activation through anti-tumor hCD19 CAR-T or both αhCD19 BsTE:CAR-T and αPD-L1 BsTE:CAR-T cell treatment in hCD19 MC38-transplanted mice was evaluated by measuring the released INF-γ levels of CAR-T cells. Both hCD19 CAR-T and αhCD19 BsTE:CAR-T cells showed similar INF-γ levels, whereas αPD-L1 BsTE:CAR-T cells showed significantly increased levels (Fig. 7E).

To investigate the migration of hCD19 CAR-T cells by αhCD19 BsTE or αPD-L1 BsTE in vivo, C57BL/6 mice were transplanted with MC38 and hCD19 MC38. Both types of tumor cells (2 × 10^6^ cells/mouse) were transplanted, MC38 in the left flank and hCD19 MC38 in the right flank. Ten days after initial tumor cell transplantation, 1 × 10^6^ hCD19 CAR-T, αhCD19 BsTE:CAR-T, or αPD-L1 BsTE:CAR-T cells were intravenously administered into the double tumor bearing mice to evaluate their migration into tumors. After 2 days from the injection of hCD19 CAR-T cells, the mice were euthanized and tumor infiltration of hCD19 CAR-T cells was analyzed. As expected, in the double tumor-bearing mice, αPD-L1 BsTE:CAR-T cells showed significantly upregulated migration toward hCD19 MC38 tumors compared with that in the control, hCD19 CAR-T-, and αhCD19 BsTE:CAR-T cell-treated group, whereas no significant differences in migration toward MC38 tumors were observed (Fig. 7F). Furthermore, immunofluorescence analysis showed that αPD-L1 BsTE:CAR-T cell treatment yielded a higher infiltration of hCD19 CAR-T cells into tumor tissues than that in the other groups (Fig. 7G, H). These results suggest that αPD-L1 BsTE engagement with hCD19 CAR-T cells enhances the infiltration of hCD19 CAR-T cells into tumor tissues and the killing activity of CAR-T cells. This may be because previous results suggest that αPD-L1 BsTE shows anti-tumor activity in a PD-L1 dependent-manner because of PD-L1^+^ exosomes, whereas hCD19 BsTE does not.

## Discussion

This study aimed to verify the efficacy and anti-tumor mechanisms of αCD3 × αPD-L1 BsTE:T in vitro and in vivo. The results showed that BsTE:T administration effectively inhibited cancer cell growth more than BsTE or activated T-cells alone did in an in vivo syngeneic mouse model and a human multiple myeloma model in NOG mice. These results were closely linked to the increased migration of T-cells into cancer tissue. The migration of T-cells to the tumor site was closely related to the expression of PD-L1 protein, the cancer cell surface target of BsTE molecules, and PD-L1-containing exosomes secreted by cancer cells. Moreover, bystander CD8^+^ T-cells without tumor antigen specificity, i.e., CD44^+^CD8^+^ T-cells, contributed to the elimination of tumor cells [22]. Overall, this study provides insights into the potential of αCD3 × αPD-L1 BsTE:T as novel anti-cancer therapeutic agents and reveals the underlying mechanisms of migration and trafficking of BsTE:T in vivo.

The concept and efficacy of killing cancer cells using bispecific antibodies and activated T-cells has been known since the late 1980s, whereby quadroma antibodies or chemically conjugated bispecific antibodies were used simultaneously with activated T-cells for specific delivery to cancer cells [23–25]. With advances in antibody engineering, humanization, and screening technology, human antibody-based bispecific antibodies have been actively developed in recent years [26]. In particular, several attempts have been made to combine the specific engaging functions of bispecific antibodies with the immunological activities of several immune cells. One such development is the bispecific antibody-armed activated T-cell strategy, comprising ATCs with αCD3 × anti-TAA BsAb, which converts ATC into non-major histocompatibility complex-restricted anti-tumor cytotoxic T lymphocytes [27, 28]. The binding of target antigens via the BsAb bridge enables specific anti-tumor cytotoxicity, Th1 cytokine release, and T-cell proliferation [29, 30]. Clinical trials in breast, prostate, and pancreatic cancers using ATCs have demonstrated safety, feasibility, induction of anti-tumor immune responses, and potential increases in overall survival [28]. However, no detailed studies on the basic mechanisms underlying the improved anti-cancer activities of bispecific antibody-armed activated T-cell treatment have been conducted to date.

In our study, we observed higher BsTE-bound T-cell infiltration into tumor tissue than that of T-cells alone and confirmed that some of the infiltrated T-cells were indeed bound to BsTE. These results suggest that BsTE on the surface of T-cells is involved in a molecular mechanism that encourages the enhanced migration of T-cells into the tumor, providing a basis for high tumor-killing rates. Through a competition experiment with an αPD-L1 antibody, PD-L1-containing materials appeared to be involved in BsTE:T migration, for which exosomes from cancer cells were selected. GW4689 treatment confirmed the involvement of exosomes in BsTE:T infiltration.

From the BsTE:T migration experiment with the collagen semipermeable layer, in which PD-L1 antibody treatment abrogated T-cell migration, either PD-L1 on a membranous particle or shredded extracellular PD-L1 could have affected T-cell movements. However, GW4869, a pharmacological agent that blocks exosome production, inhibited BsTE:T migration, suggesting that PD-L1-containing exosomes could be key mediators between T-cell migration and PD-L1 function. This mechanism may draw BsTE:T closer to cancer cells through PD-L1 gradient-containing exosome particles, which were confirmed in the cancer cell lines and patient samples in the present study (Fig. 3).

Generally, PD-L1-containing exosomes are referred to as immunosuppressants [31, 32]; however, in the current study, they were involved in the migration and activation of T-cells. When PD-L1 molecules on the surface of cancer-origin exosomes bind to PD-1 on activated T-cells, it may result in the suppression of T-cell activity. However, when activated T-cells are pre-treated with bispecific antibodies, especially αPD-L1 and CD3, exosomal PD-L1 can function differently, augmenting T-cell receptor activation signals on T-cells via BsTE bridging activity.

PD-L1-dependence in vitro and in vivo, tumor infiltration, cytokine release, BsTE:T killing activity, and IFN-γ production, in addition to PD-L1^+^ exosomes on the PD-L1 KO tumors (MO5 and MC38), suggest that tumor-derived exosomes with tumor antigens play an important role in the enhancement and infiltration of BsTE:T.

BsTE exerts its activation function only after it reaches the tumor site and bridges tumor and T-cells via fundamental mechanisms that have been previously described [33, 34]. Furthermore, the release of Th1 cytokines during infiltration is unclear; however, the involvement of tumor-derived exosomes containing TAAs as active components may complete our understanding of the enhanced tumor-killing and infiltration mechanisms associated with BsTE-bound activated T-cells.

The physiological role of PD-L1 present in exosomes may be associated with the migration of regulatory T-cells, which are commonly present in the tumor microenvironment where they exert their immunosuppressive role. In our preliminary experiments, the exosome inhibitor GW4869 abrogated the migration of regulatory T-cells in vitro (data not shown). Therefore, the BsTE:T strategy that shifts the infiltration balance between effector and regulatory T-cells has additional advantages, wherein cell adhesion molecules highly enriched in small extracellular vesicles promoting cell adhesion during migration may play additional roles in enhancing immune cell migration[35, 36].

The important role of PD-L1 antibody in the BsTE construct has not been fully elucidated; however, its function in converting diverse T-cells into non-major histocompatibility complex-restricted anti-tumor cytotoxic T lymphocytes could be applied to other tumor-specific antibodies. However, because PD-L1 can be regarded as a tumor specific-antigen and an immune checkpoint molecule, αPD-L1 antibody may have a dual role in the BsTE construct: one as a tumor-T-cell engager and the other as an immune checkpoint blocker. Future comparisons with other BsTEs targeting different antigens, such as Her-2 or epidermal growth factor receptor, will help elucidate the mechanism underlying the enhanced tumor-killing effects of BsTE:T.

Compared with BsTE alone and the co-administration of BsTE and T-cells, ex vivo BsTE:T exhibited superior efficacy. Regarding the fast clearance of BsAb based on tandem double scFvs in the bloodstream, the occurrence of activated T-cells charged with BsTE is not prevalent enough to provide sufficient anti-tumor activity in vivo. However, ex vivo binding of BsTE to pre-activated T-cells can provide sufficient BsTE:T density and surface BsTE molecules to mediate successful anti-tumor immunity in vivo. BsTE binding on the T-cell surface, which is affected by the characteristics of BsAb, is reportedly highly stable, lasting approximately 83.8–135.4 h [37].

In conclusion, this study investigated the trafficking and migration of BsTE:T to improve the efficacy of BsTE in eliminating cancer cells. BsTE:T eliminated cancer cells more efficiently than T-cells alone did. Exosomes and PD-L1 expression in tumors play critical roles in BsTE:T migration and anti-tumor activity. Bystander CD8^+^ T-cells also contribute to the elimination of tumor cells. This study provides insights into the potential of αCD3 × αPD-L1 BsTE:T as a novel anti-tumor therapy and helps elucidate the mechanisms underlying the trafficking and migration of BsTE:T in vivo.

## Supplementary Information

Below is the link to the electronic supplementary material.

Supplementary file1 (DOCX 12 kb)

## Data Availability

Further information and requests for resources and reagents should be directed to and will be fulfilled by the lead contact, Hyun-Jeong Ko (hjko@kangwon.ac.kr).
