# Supplementary material for: The expression of PD-L1 on tumor-derived exosomes enhances infiltration and anti-tumor activity of αCD3 × αPD-L1 bispecific antibody-armed T cells
Source: Cancer Immunol Immunother. 2024 Aug 6;73(10):196. doi: 10.1007/s00262-024-03785-4 (PMC11303351; doi:10.1007/s00262-024-03785-4)

**Supplementary figure captions**

**Supplementary Fig. 1** Generation of bispecific T-cell engager (BsTE)-bound T-cells confirmed using an antibody against c-Myc, which is one of the structures of BsTE

**Supplementary Fig. 2** (A, B) Cluster of differentiation (CD)8^+^ T-cells were obtained from the spleen and lymph nodes of C57BL/6 mice. The activated group received anti-CD3/CD28 antibodies for 48 h, and the bispecific T-cell engager (BsTE) group was incubated with BsTE (0.1 μg/mL) for 1 h after activation. ns; not significant and *****p* < 0.0001 (one-way ANOVA; *n* = 3/group). (C) Naive CD8^+^ T-cells obtained from C57BL/6 mice were cultured with or without anti-CD3/CD28 antibodies and stained with an anti-CD3 antibody to measure the level of CD3 expression in CD8^+^ T-cells

**Supplementary Fig. 3** T-cells or bispecific T-cell engager-bound T-cells (BsTE:T) were activated with anti-cluster of differentiation (CD)3/CD28 antibodies for 48 h and BsTE (0.1 μg/mL) cells were incubated for 1 h. T-cells or BsTE:T cells were seeded on top of the transwell and MO5 cells were seeded at the bottom of the transwell. Thereafter, the experimental group was treated with GW4869 and incubated for 24 h. ns, not significant, **p* < 0.05, and ***p* < 0.01 (one-way analysis of variance; *n* = 6/group)

**Supplementary Fig. 4** (A, B) The number and population of cluster of differentiation (CD)3^+^CD8^+^CD44^high^CD62L^high^ central memory T-cells in tumor-infiltrated lymphocytes of each group of mice were analyzed using fluorescence-activated cell sorting (FACS). ns, not significant (one-way analysis of variance; *n* = 8/MC38, *n* = 5/PD-L1-knockout MC38).

**Supplementary Fig. 5** (A, B) Bispecific T-cell engager molecules were generated using single chain fragment variables from α cluster of differentiation (CD)3ε (OKT3) and α programmed death-ligand 1 (PD-L1) antibodies (KL001-13) and were linked with the ‘GGGGS’ linker. V_L_/VL, variable light chain; V_H_/VH, variable heavy chain; hBsTE, human BsTE


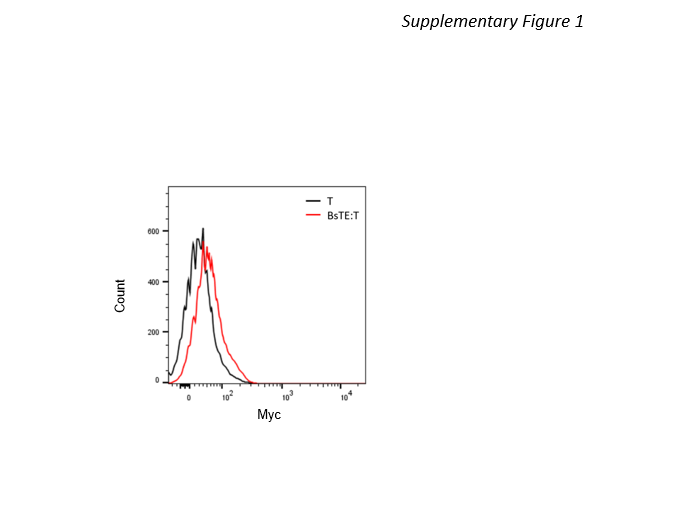

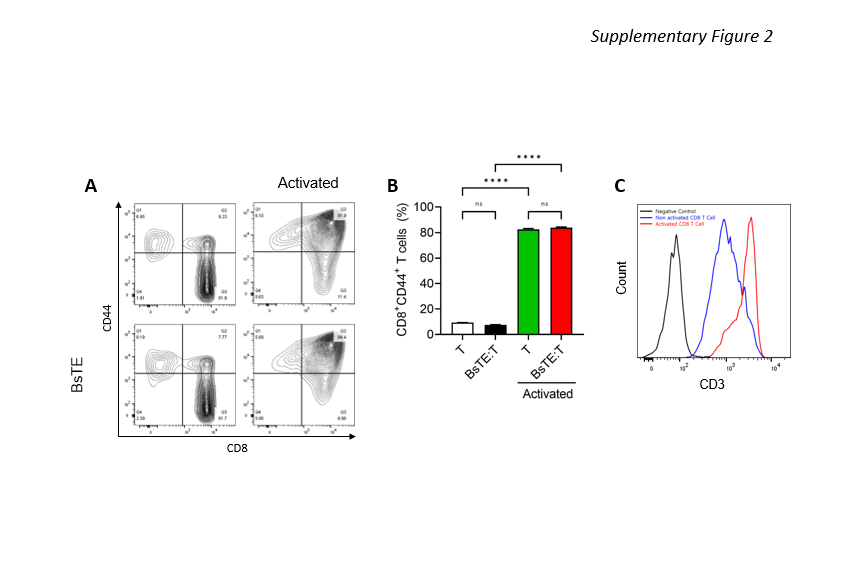

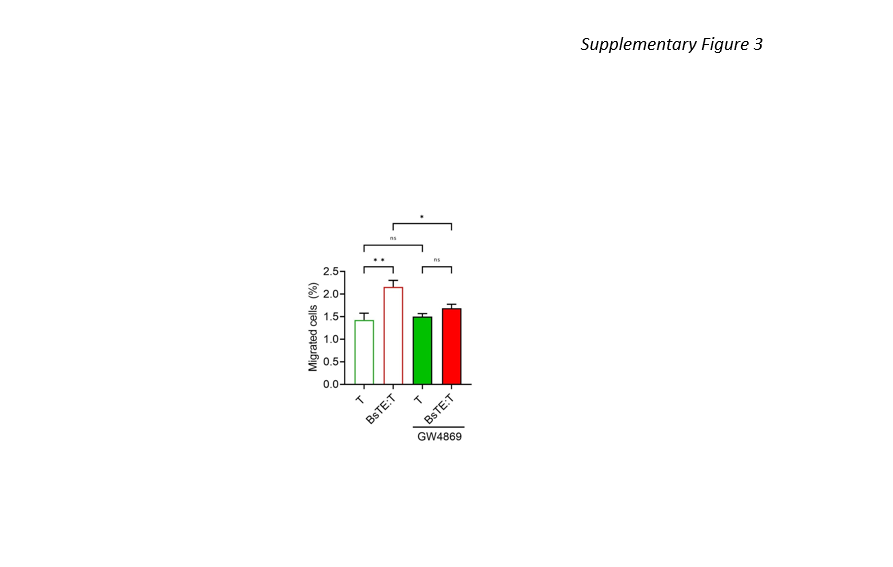

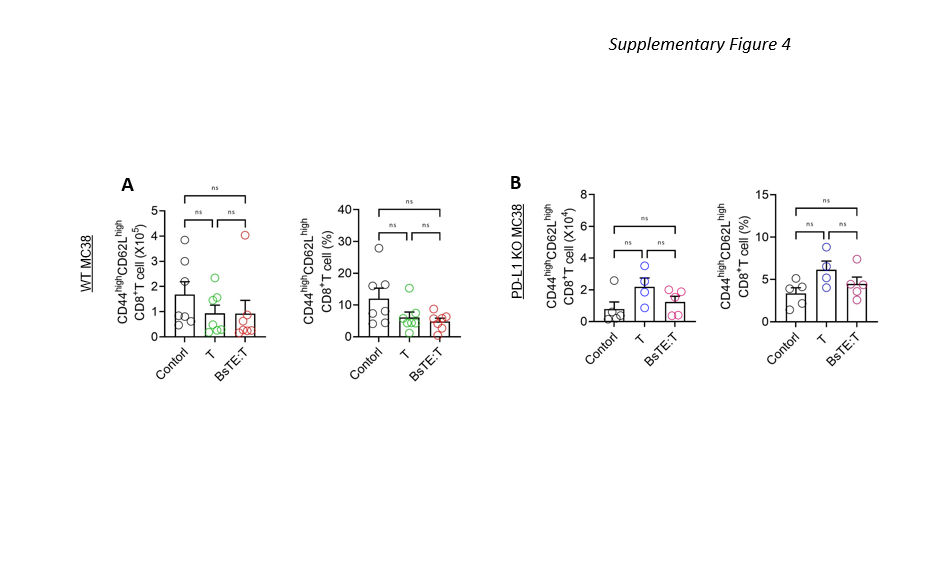

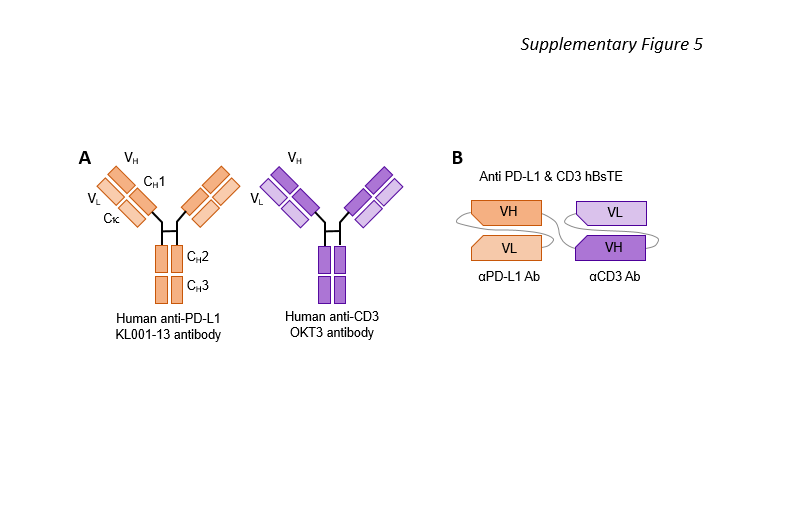

Supplement: Supplementary file 1 — Supplementary file1 (DOCX 12 kb) [file 262_2024_3785_MOESM1_ESM.docx]
